# How To Record a Million Synaptic Weights in a Hippocampal Slice

**DOI:** 10.1371/journal.pcbi.1000098

**Published:** 2008-06-20

**Authors:** Upinder S. Bhalla

**Affiliations:** National Centre for Biological Sciences, Tata Institute of Fundamental Research, Bangalore, India; Indiana University, United States of America

## Abstract

A key step toward understanding the function of a brain circuit is to find its wiring diagram. New methods for optical stimulation and optical recording of neurons make it possible to map circuit connectivity on a very large scale. However, single synapses produce small responses that are difficult to measure on a large scale. Here I analyze how single synaptic responses may be detectable using relatively coarse readouts such as optical recording of somatic calcium. I model a network consisting of 10,000 input axons and 100 CA1 pyramidal neurons, each represented using 19 compartments with voltage-gated channels and calcium dynamics. As single synaptic inputs cannot produce a measurable somatic calcium response, I stimulate many inputs as a baseline to elicit somatic action potentials leading to a strong calcium signal. I compare statistics of responses with or without a single axonal input riding on this baseline. Through simulations I show that a single additional input shifts the distribution of the number of output action potentials. Stochastic resonance due to probabilistic synaptic release makes this shift easier to detect. With ∼80 stimulus repetitions this approach can resolve up to 35% of individual activated synapses even in the presence of 20% recording noise. While the technique is applicable using conventional electrical stimulation and extracellular recording, optical methods promise much greater scaling, since the number of synapses scales as the product of the number of inputs and outputs. I extrapolate from current high-speed optical stimulation and recording methods, and show that this approach may scale up to the order of a million synapses in a single two-hour slice-recording experiment.

## Author Summary

The circuitry of the brain is defined by the connections (synapses) between its cells. Synapses are very small, so it is difficult to identify more than a few at a time using standard methods like electron microscopy or high-precision electrical recordings from cells. This study shows that it is possible to measure single synapses using low-precision methods such as optical recordings from neuronal cell bodies. I model optical or electrical stimulation of many inputs to trigger a visible response from neurons, and find single synapses by testing how this response is modulated when a single additional input synapse is triggered as well. I predict that it should be possible to record from as many as a million synapses using new optical recording and stimulation methods. It is believed that memories are encoded in synaptic connection patterns, so such connectivity data may give us a picture of how memories are encoded. We now know a great deal about how individual neurons behave, so a synapse-level wiring diagram would go a long way to fill out the picture of how neurons work together in the brain to interpret sensory information and plan actions.

## Introduction

**Figure 1:**
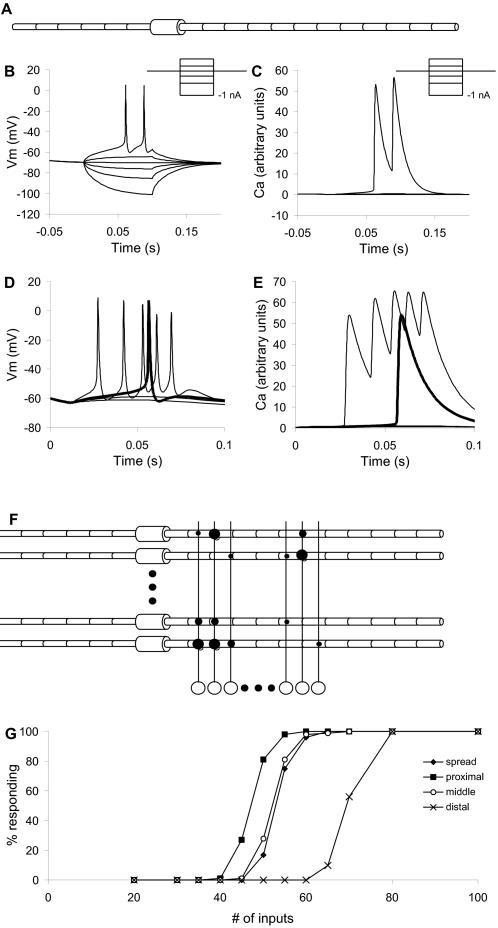
Basic Model. (A) Compartmental structure of CA1 neuronal model. The model had 19 compartments, including 12 apical dendritic compartments and one soma. (B) Somatic intracellular potential responses of compartmental model to current injection pulses from −1 nA to +0.5 nA (inset). (C) Ca^2+^ responses to same current series. (D) Somatic intracellular potential responses to synaptic input on 40, 50, 60 and 100 identical synapses, delivered at t = 10 ms. Input to 60 synapses elicited one action potential, plotted in bold. (E) Corresponding Ca^2+^ responses. (F) Schematic of network model structure. There were 100 CA1 neuronal models and 10000 single-compartment input neuron models. The different synaptic strengths are indicated with different sized circles. Note that the connection probability was 5%, so the actual connectivity was much sparser than shown. (G) Responses of a population of simulated neurons to different numbers of inputs distributed over the neuron. The synaptic weights were modeled as a Gaussian distribution as described in the Methods. The ‘spread’ input case had about 50% responses for 50 inputs.

The neuronal wiring diagram of many mammalian brain regions is known in a statistical sense, but not at the level of individual neurons [1]. The hippocampal CA3 to CA1 region is a particularly simple circuit with considerable functional relevance in memory, and is therefore an interesting test case for working out detailed connectivity. An idealized way to work out the neuronal connection matrix (Figure 1F) is to stimulate one input neuron at a time, record the outputs of each CA1 neuron, and enter these values as the weights of that row of the connection matrix. The major exercise of this paper is to analyze how to detect individual synapses, despite experimental limitations that complicate this idealized approach.

The first limitation is stimulus specificity. How can we stimulate exactly one input neuron at a time? Recent optical stimulation experiments using localized glutamate uncaging [2]–[4] have been used to estimate spatial connectivity profiles in the hippocampus and cortex. These studies provide high spatial resolution, which approaches single neuron resolution. Optogenetics provides another approach [5],[6]. By inserting the channelrhodopsin-2 (ChR2) gene into hippocampal neurons, it is possible to stimulate cells with <5 ms precision [7]. Current ChR2 constructs have not been reported to be used with 2-photon excitation to obtain single-neuron specificity in the slice, but the method does provide for genetic targeting to specific neuronal populations (reviewed in [8]). High resolution is also possible using minimal stimulation on arrays of electrodes [9], though it is difficult to scale this to more than a few hundred inputs.

The second limitation is output sensitivity. Whole-cell patch recordings have long been used as sensitive measures of synaptic responses. Modeling and experimental studies have used patch-clamp data in the presence of spontaneous activity to obtain distributions of synaptic conductances [10],[11]. When coupled to electrical [12],[13] or optical [14] stimulation, patch recordings have been used to obtain spatial distributions of synaptic connectivity in networks. Individual synaptic data are more difficult to obtain in neural circuits. By performing large numbers of pair wise patch-clamp recordings it is possible to measure single synaptic connections in circuits (e.g., [15]). As many as seven cells have been reported to be patched simultaneously (e.g., [16]), and the current technical limit is ∼12. However, patch recordings do not scale well. Demanding experiments such as multi-patch recording can give connectivity information for tens of synapses, but one would like to work out circuitry for many times this number, and to do so in an individual slice. The alternative is to turn to more scalable but less sensitive methods. These include Ca^2+^ recordings and single-unit extracellular electrode recordings.

Ca^2+^ dye recordings have been used to simultaneously monitor hundreds of individual neurons [17]. Implanted extracellular electrodes have also been used to record from hundreds of isolated single units [18],[19]. Such recordings require the neuron to fire and generate a Ca^2+^ transient or an extracellular spike. This is a highly nonlinear process. Even with intracellular recordings, spiking nonlinearities greatly complicate the estimation of synaptic conductances [20]. It typically takes 50 or more simultaneous synaptic inputs to elicit an action potential in CA1 pyramidal neurons [21],[22]. In other words, we would not be able to see a response to a single input fiber stimulus. In this study we overcome this by adding a baseline stimulus to bring the output neuron near threshold, and then monitor how the addition of the single fiber stimulus changes the response.

The third limitation is stochasticity in synaptic release [23],[24]. Even with perfect optics and recordings, there is a modest (50% or smaller) probability that any given action potential will elicit a postsynaptic response at a given synapse. Furthermore, this probability is strongly history dependent. This introduces variability in neuronal responses to identical stimuli. While this variability complicates estimates of synaptic connection strength, it is also an essential requirement for the proposed method. Stochasticity in synaptic release helps the measurement by providing fluctuations near the action potential threshold, so that repeated samples reveal differences in distribution due to the addition of the single synaptic stimulus. A similar process has been proposed for physiological neuronal responses in the context of subthreshold background input, and has been shown to be effective in improving detection of single synaptic inputs [25].

In this study I perform a series of *in silico* experiments on the hippocampal CA1 network to design and analyze a synaptic estimation method that only needs extracellular or low-resolution optical recordings. These computational ‘experiments’ have the advantage that the correct synaptic weight matrix can be directly read out from the model definition, as well as from more physiologically practical readouts such as Ca^2+^ responses. This gives an unambiguous assay of the accuracy of synapse prediction. The method scales as the product of number of stimulus points and readout neurons. With current techniques this method should be able to resolve thousands of synapses, and it has the potential to scale to around a million.

## Results

I simulated hippocampal slice optical recording experiments designed to obtain synaptic weight matrices. The basic design of these experiments was to deliver a background stimulus to a block of Schaffer collaterals or CA3 neurons so as to bring postsynaptic CA1 cells above firing threshold. A probe stimulus was delivered to a single input neuron, over this background stimulus. By comparing responses to background and background+probe stimuli, the presence and potentially the strength of synaptic connections could be determined. In principle the background stimulus could be delivered directly to the output neurons using ChR2 or glutamate uncaging, but this seemed unnecessarily complex because the input axons/CA3 neurons would already be set up for the stimulation procedure.

I first calibrated the basic properties of the models. Then I explored different contributions to noise in the network and readouts. Finally I simulated a set of complete experiments including multiple sources of noise and several variants on the full network, to obtain an experimental and analysis design capable of reading the wiring of a large network.

### Simulation Calibration

I simulated 10000 input fibers and 100 CA1 output neurons with 19 compartments, multiple channel types, and Ca^2+^ dynamics (See Methods and Figure 1). The model size was determined by two considerations. First, many axons must be stimulated to elicit detectable Ca^2+^ responses. Assuming 5% connectivity, and a requirement for ∼50 simultaneous inputs, we would need 50/0.05 = 1000 axons to trigger an output action potential (Figure 1G). In order to have a reasonable number of such sets, the simulation used 10,000 input fibers. Second, the calculations needed a representative sample of output neuron properties, and enough output neurons that the random synaptic connectivity would form a representative distribution of inputs.

### Noise-free Simulations

As a first pass approach to testing the feasibility of the approach, I modeled the 10000-axon, 100 neuron network without noise and with identical neurons (Figure 1F). I delivered inputs to a group of 1000 consecutive input fibers, numbered 1 to 1000. I selected one more fiber on which to deliver the probe stimulus in addition to the background 1000 inputs. I then advanced the entire stimulus set by 1 axon, so that the input block was from 2 to 1001. The probe stimulus was also advanced by 1 axon. I repeated this process for the entire set of 10,000 axons, so that all axons had been probed individually, and had contributed to the background 1,000 times.

To find the synapses I compared the response to background (RB) to the response to the background+probe (RP). Whenever RP>RB, it was inferred that a synapse was present between the probe axon and neuron. This was used to build up a synaptic connection matrix (Figure S1). This matrix was accurate but missed a few synapses (170 out of ∼50,000) because of premature truncation of the Ca^2+^ signal in the simulations. However, in the presence of as little as 0.5% Gaussian readout noise this method was completely inaccurate (Figure S1).

### Contributions to Noise

I considered four sources of noise in the experimental system:

1. Variability between cells in the network (intrinsic variability)
2. Baseline activation noise due to synaptic release probability (input noise).
3. Probe noise due to synaptic release probability (input noise)
4. Readout noise in the optical recording system (output noise).

Notably, the first three are inherent properties of the biological system and were incorporated into the model (Methods). Only the readout noise is under the technical control of the experimenter. It was added at analysis time.

**Figure 2:**
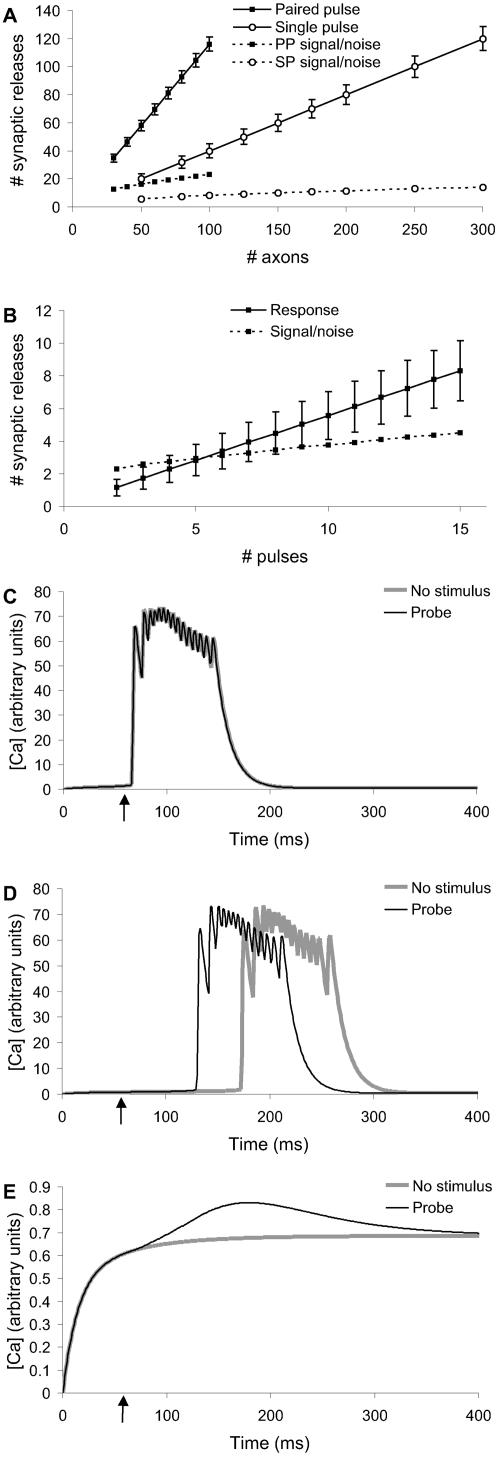
Designing the Stimulus Protocol. (A) Baseline stimulus distribution scaling with number of axons. Paired pulse stimulus had better signal-to-noise and required fewer input axons than single pulse. (B) Probe stimulus scaling with number of pulses. Signal-to-noise (on same axis) improved slowly. (C, D, E) Comparisons of baseline (no stimulus) and baseline+probe responses at −65, −66 and −67 mV reversal potential for potassium. The curves were separable only at −66 mV and even at this potential the cells went into spontaneous bursts.

In initial Monte Carlo calculations I computed the noise arising from probabilistic synaptic release for the baseline stimulus (Dataset S1, Figure 2A). I used estimates for single and double action potential releases [23], with the following parameters: probability of release on first pulse = 0.4, on subsequent pulses if no earlier release = 0.9, and on subsequent pulses after an earlier release = 0.55. While this was a simplistic model and did not account for all forms of synaptic and release variability (e.g.,[24],[26]), it did result in considerable stochasticity. The calculations showed that the signal-to-noise ratio was substantially better for paired action potentials (Figure 2A). Therefore the baseline stimulus protocol was designed to use paired pulses.

In a similar manner, I computed the distributions of actual number of inputs as a function of number of pulses in the probe stimulus (Figure 2B). There was a large standard deviation of ∼35% for 6 pulses.

### Synapse Probing Using KCl Depolarization

Instead of using baseline stimuli, an alternative approach could be to use KCl to raise the cellular resting potential near threshold [13]. In principle, probe stimuli riding on a near-threshold depolarization should be able to elicit action potentials and Ca^2+^ transients. I modeled this experiment by altering the reversal potential of all modeled K+ channels, and depolarizing the membrane potential Em in all compartments in the neuronal models. The modeled network incorporated cellular variability and probe synaptic release variability. These simulations showed that probe stimuli elicited responses only in a very narrow window of ∼1 mV resting potential (Figure 2C, D, E). Above this window cells tended to go into bursting activity. Because of variability between cells, this window differed between cells. Thus, at least for CA1 pyramidal neuron physiology, KCl depolarization did not appear to be a viable approach.

### Stimulus Pattern Optimization

**Figure 3:**
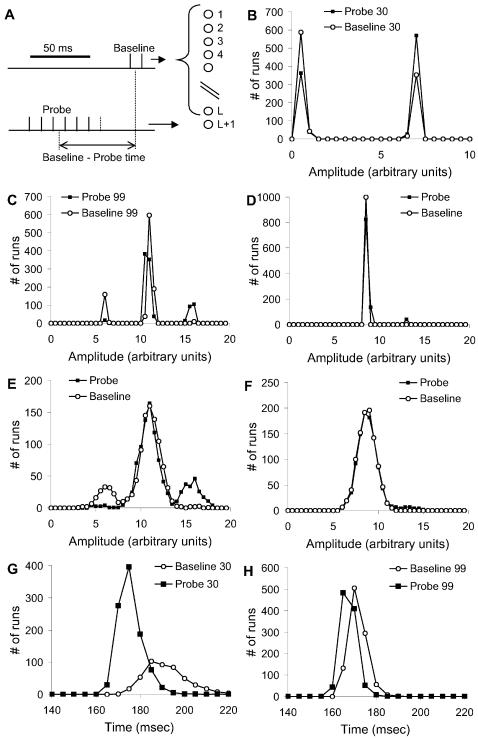
Refining the Stimulus Protocol. (A) Stimulus protocol. L axons were stimulated for the baseline stimulus, using two pulses. The L+1 axon was used for the probe stimulus, consisting of N pulses at varying times before the baseline stimulus. The reference condition used L = 1300, N = 6 and probe-baseline = 30 ms. (B, C) Distributions of Ca^2+^ response amplitudes for neurons 30 and 99 respectively. The responses were clumped into a few bins, corresponding to the number of action potentials elicited by the stimulus. (D) Distribution for neuron 99 without baseline stimulus stochasticity, but with probe stimulus stochasticity. A small number of baseline+probe runs had a second action potential. (E) Same responses as (C) with 20% gaussian instrumentation noise. The side peaks were still clearly visible and the probe+baseline distribution was easily separated from baseline. (F) Same responses as (D) with 20% instrumentation noise. The side peak was almost obscured and the two distributions were hard to separate. (G, H) Distributions of Ca^2+^ response timing for neurons 30 and 99 respectively. There was a 10 ms peak difference for neuron 30, and a 5 ms difference for neuron 99.

To design effective stimulus patterns, I performed a series of simulations to characterize the distributions of responses without and with probe, RB and RP. I used only a single probe position, but carried out the RB and RP simulations 1000 times each. These runs excluded instrumentation noise but included probabilistic synaptic release and cell-to-cell variability. The simulations used 1300 baseline axons, and 6 pulses for the probe stimulus (Figure 3A).

I first assessed which of two readouts of neuronal responses (Ca^2+^ amplitude and timing) were most informative. I examined the raw distributions of RP and RB for sample neurons that were known from the model definition to be connected to the probe axon. As expected from spike-triggered Ca^2+^ influx, the amplitude responses were clustered into a small number of bins (Figure 3B, C). Similar multi-peak distributions have also been seen experimentally (Parameshwaran and Bhalla, unpublished data). RP distributions had more samples with larger responses, indicating that the probe stimuli occasionally elicited additional action potentials. If baseline synaptic noise was eliminated from the simulations, the difference in distributions was much smaller (Figure 3C–F). This suggested that the difference in distributions was amplified by stochastic resonance.

In the case of the timing responses, there was a small shift of 5–10 ms in the position of the RP vs. the RB distribution (Figure 3G, H). 10 ms is at or below the resolution of most Ca^2+^ recording methods, due to slow kinetics of most dyes. However, electrical recordings such as extracellular recordings, have <1 ms time resolution and would be well suited to using timing data. The current analysis is restricted to optical readouts and therefore does not use timing data.

**Figure 4:**
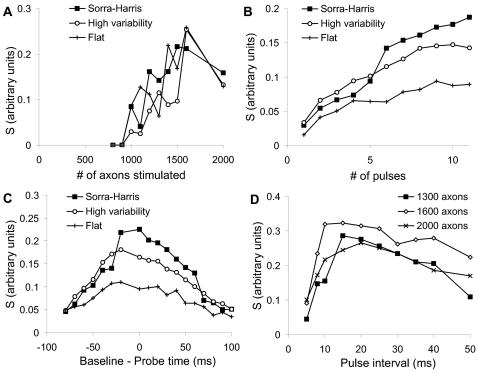
Separation of Responses. Separation S is calculated for three different conditions in (A, B, C): Sorra-Harris synaptic weight distribution with 20% variability between cells, a high (50%) cell-to-cell variability case again using the Sorra-Harris distribution, and a flatter distribution truncated to have the same mean as the Sorra-Harris distribution, also with a 20% variability. (A) Separation S as a function of L, the number of axons stimulated for the baseline. (B) S as a function of N, the number of probe pulses. (C) S as a function of probe-baseline time. (D). S as a function of interval between pulses in the probe and baseline stimuli, for values of L = 1300, 1600 and 2000 axons.

I performed an initial survey of the amplitude data using a separation measure S based on means and standard deviations:
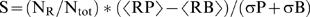
(1)Here N_R_ is the number of responding neurons, N_tot_ the total, 〈RP〉 and 〈RB〉 are the means of RP and RB, respectively, and σP and σB are the standard deviations of their distributions. This measure was not optimal given the strongly non-Gaussian nature of the response, but was useful for an initial characterization of the data. I considered four parameters that could affect the separation between responses to baseline and probe stimuli:

1. Number of axons in background set (L). I computed S for different values of L. I used 6 probe pulses and a probe time lead of 30 ms. S rose with L, but showed a decline over L = 1600. (Figure 4A). I selected L = 1300 as a good intermediate value for further calculations.
2. Number of pulses in probe stimulus. L was fixed at 1300 axons, and the end of the probe sequence coincided with the start of the baseline stimulus. As expected, separation improved with larger number of pulses (Figure 4B). As a compromise between increased separation and the duration of the stimulus protocol, I used 7 pulses for further analysis.
3. Timing of probe stimulus with respect to background. L was fixed at 1300 axons and the number of pulses was 7. The response was maximal if the probe stimulus straddled the background stimulus (Figure 4C). I interpreted this as being due to rapid charge decay.
4. Pulse interval. Again, 7 pulses were used, such that they straddled the background stimulus. Three values of L were tested: 1300, 1600, and 2000 axons. The pulse interval was varied between 5 and 50 ms for both the baseline and the probe stimuli. To do so I set the synaptic release probability as a function of inter-pulse interval according to published data [23]. I found that the optimal pulse interval varied with L, and was best between 10 and 20 ms inter-pulse interval (Figure 4D). At smaller intervals, the synaptic release probability was low for the second pulse, and this pulled down the sensitivity. At longer intervals the electrical decay of the synaptic input also caused a decrease in sensitivity. I selected an interval of 10 ms for further analysis.

Overall the best value of separation S was ∼0.25. As a rough estimate, this should improve as √N, where N is the number of repetitions. The target accuracy is 0.05% errors, to achieve an error of less than 1 in 100 of connected synapses, which in turn are 5% of total possible connections. This requires around 4 standard deviations. If the baseline variability σB can be eliminated, the separability requirement is halved, so we would need a total of ∼64 repetitions.

### Designing a Stimulus Protocol

**Figure 5:**
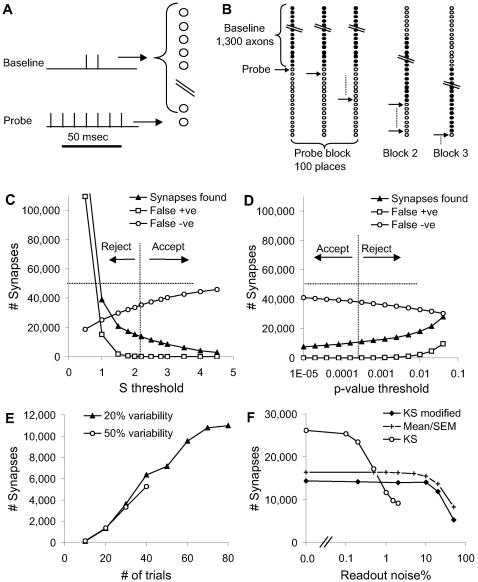
Finding Synapses From the Full Network. (A) Stimulus design in time. The baseline stimulus had two pulses. The probe stimulus had seven pulses that straddled the baseline. (B) Stimulus design across axons. The baseline stimulus consisted of 1300 axons. The position of the baseline remained the same for 100 different positions of individual probe stimuli. This was referred to as a block of baseline/probe stimuli. Then the baseline was shifted over by 100 positions, and a new block of baseline/probe stimuli was delivered, and so on. (C) Mean/SEM statistic S used to classify synapses. The classification was accepted (vertical dashed line) only if the number of false positives was less than 1% of the number of classified synapses. At high values of S very few synapses were classified at all, and the number of false negatives approached the total number of synapses (horizontal dashed line). (D) Similar classification using the modified Kolmogorov-Smirnov probability p. This statistic correctly classified fewer synapses. (E) Modified KS classification as a function of # of stimulus repeats. The same 1/100 false positive criterion was applied. The results were only slightly affected by variability between neuronal parameters. (F) KS and mean/SEM classification as a function of instrumentation noise (simulated as Gaussian noise with mean zero and the specified standard deviation). The KS statistic did better at low noise, whereas mean/SEM and modified KS methods worked even for high noise levels.

Based on these data, I designed a stimulus procedure to resolve synapses. This stimulus design is shown schematically in Figure 5A, B. A movie of the stimulus applied to a reduced version of the network is shown in Video S1. The key features of this stimulus were as follows:

- The background input was given in blocks of size B ( = 100 in these simulations). This meant that 13 blocks were used for 1300 axons. It also meant that probe stimuli from 1301 to 1400 all used the same stimulus blocks, and so on for the next 100 probe stimuli. The use of stimulus blocks was meant to address two experimental constraints: delivering stimuli to large groups of axons, and interleaving stimulus blocks to reduce plasticity. Large groups of axons are much easier to stimulate in blocks using a high-current pulse on a single electrode, or a broad spot of light, than by individually stimulating individual neurons or axons. Plasticity issues are considered below. The probe input consisted of 7 pulses straddling a paired-pulse baseline stimulus. All pulses were 10 ms apart.
- I did not deliver separate baseline stimuli. Instead I used the entire dataset of all background+probe responses within a given background block, as the baseline. The reasoning was that for any given probe, only 5% of target neurons would receive input. Thus the distribution of all the background+probe inputs should be close to the true background input.
- I repeated the stimuli many times for each probe position.

This total stimulus set was very large, requiring 800,000 stimuli for 80 trials per probe position. If we were to deliver 3 stimuli per second this would take ∼67 hours. Later I discuss how to reduce this to experimentally feasible durations.

### Analysis

I looked for differences between baseline and baseline+probe responses using two methods: standard errors (mean/SEM test), and a variant on the Kolmogorov-Smirnov (KS) test (see Methods). Each of these measures was able to resolve ∼10,000 synapses out of ∼50,000 in the network, with <100 false positives (Figure 5C, D). These estimates were for a Sorra-Harris distribution of weights [27], with 20% neuronal variability and 20% instrumentation noise. Although the mean/SEM test could resolve slightly more synapses with optimal threshold settings, these settings were not as consistent across noise levels and repeats as the p-value of the modified KS test. Importantly, this p-value enabled an estimate of the number of false positives (Methods).

I tested how the number of identified synapses scaled with the number of trials, as this was a key consideration in the experimental design (Figure 5E). As expected, there was a steady improvement in numbers. If the neuronal population had 50% cell-to-cell variability, this caused only a small reduction in the ability of the KS test to classify synapses. I then considered how experimental readout noise affected the number of classified synapses (Figure 5F). Increased noise degraded classification, but the falloff was graceful. Classification was ∼20% of synapses for 80 repetitions, for experimentally achievable noise levels of 10 % to 20%. For extremely low noise levels the original KS test was able to classify over 50% of synapses, but it failed with even modest noise levels (Figure S2).

### Synaptic Weights

I now had a Boolean synaptic weight matrix, with 0 or 1 entries to indicate absence or presence of synapses. This led to two questions: First, was I picking up only the stronger synapses? Second, could I estimate synaptic weights?

**Figure 6:**
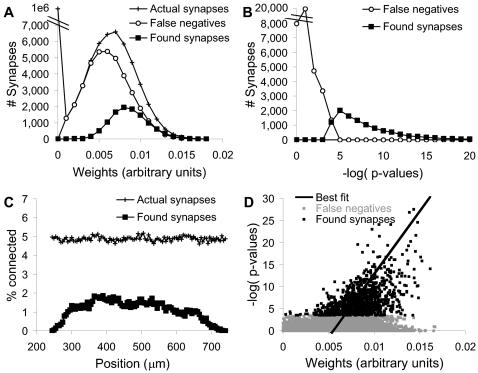
Synapse Classification Results. (A) Distribution of weights of synapses and classified synapses. The false negatives distribution had a peak below that of the found synapses, but it did include many instances of high synaptic weight. (B) Distribution of p-values of classified and rejected synapses. (C) Distribution of actual and found synapses as a function of position on dendrite. Fewer synapses were found proximal and distal to the soma. (D) Scatter plot of p-values vs. actual synaptic weights, for 20% noise, 20% neural variability and 80 repeats. Best straight-line fit for x-variance is shown.

I first examined the distribution of weights of identified synapses, and compared this with the distribution for all synapses (Figure 6A, B). I found that most of the reported synapses were strong, but that there were a substantial number of strong synapses that were false negatives. The classification success was strongly dependent on position of baseline stimulus and probe (Figure 6C), consistent with the single-neuron analysis of threshold responses (Figure 1G). It reached a peak of nearly 40% in the middle of the dendrite. This suggests that the stimulus design could be further refined to give uniformly high classification. While it was possible to fine-tune the current stimulus design, the simplicity of the CA1 pyramidal neuronal models in the current study limits the utility of such fine-tuning. A more careful analysis would require more detailed neuronal models and experimental input. There was a correlation between synaptic weight and the P-value for significance of the KS test, suggesting that synaptic weights could be estimated by this approach (Figure 6D).

### Generalizing the Method

The current method was designed for the hippocampal slice-preparation. This preparation has very little recurrence in the CA1 and very low basal activity in typical low-potassium media, and these are reflected in the simplified feed-forward design of the model. To test the applicability of the method to a broader range of neuronal circuits and experimental contexts, I considered background activity, recurrence, feed-forward inhibition, and plasticity.

I first introduced random background synaptic input to represent the use of the method in an active network context. Random synaptic activity was added at 10 Hz and 70 Hz per apical compartment in separate simulations, without modifying the existing synaptic weights. As there were 12 apical compartments, this came to 120 and 840 inputs per cell per second, respectively. The 10 Hz input did not elicit action potentials, and it actually improved synaptic resolution to nearly 36% in the presence of 20% instrumentation noise. The 70 Hz input resulted in 1–3 spikes/second in the CA1 neurons, and completely abolished the ability of the method to resolve synapses.

**Figure 7:**
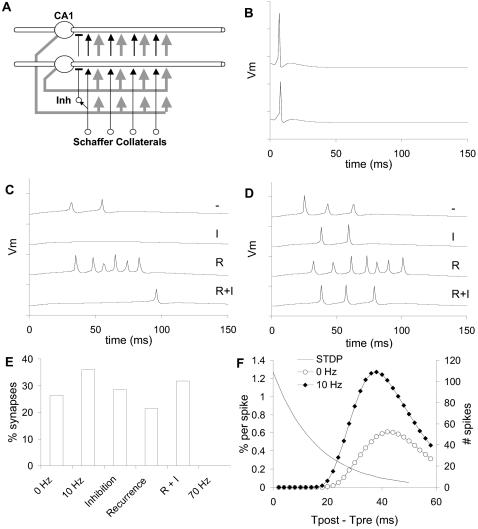
Generalization of Method. (A) Schematic of generalized circuit including feedforward inhibitory interneurons (Inh), connected to proximal dendrites of CA1 neurons; and recurrent connections from CA1 axons back to the 6 most proximal apical dendrite compartments (gray arrows). Inhibitory interneurons received inputs from ∼120 Schaffer Collateral axons, and synapsed onto ∼5 CA1 neurons. CA1 recurrent projections had a probability of ∼60% of connecting onto any one of the CA1 neurons. (B) Two example inhibitory interneuron responses. There was little variability. (C, D) Two example CA1 pyramidal neuron responses to four circuit cases. Dash: Neither recurrence nor inhibition. I: Inhibition alone. R: Recurrence alone. I+R: Both inhibition and recurrence. All the voltage traces are sampled at 1 ms so the peaks are slightly sub-sampled. (E) Synapse resolution fractions for different circuit cases. These were measured from a subset of 1000 axons out of the 10,000 in the circuit. 0 Hz: Original circuit without background activity. 10 Hz: Original circuit with 10 Hz background activation. In the Inhibition, Recurrence and R+I cases the background was fixed at 10 Hz. 70 Hz: Original circuit with 70 Hz activation leading to 1–3 Hz spiking in the CA1 neurons. No synapses were resolved in this last case. (F) STDP curves and distribution of spike timings. The smooth STDP curve has τ = 15.9 ms and A = 76% for 60 pulses, based on the fits from [28]–[30]. There was little overlap.

I next considered circuit elaborations including recurrence and inhibitory interneurons (Figure 7A) along with the 10 Hz background activity. These circuit elaborations were purely a way to introduce complications into the simple feedforward network and were not meant to be accurate models of specific biological circuits. As expected, inhibitory interneurons fired very reliably following the input volley (Figure 7B), and reduced the number of spikes that the volley elicited in CA1 neurons (Figure 7C, D). Likewise, ∼60% recurrence alone had the expected effect of eliciting a burst of action potentials, which was truncated if inhibition was also present (Figure 7C, D). Surprisingly, the synapse detection was fairly insensitive to each of these circuit elaborations (Figure 7E). There was about a 40% reduction in ability of the method to resolve synapses when recurrence was present alone and 20% for inhibition alone. These drops in synapse detection were not due to circuit complexity, but to overall excitability. When both recurrence and inhibition were present (R+I) the excitability was similar to the original model (Figure 7C, D) and the drop was only 12%. Thus the method was good at identifying first-order synapses and ignoring polysynaptic input, at least in the circuit configurations tested.

A further generalization was to consider the effects of synaptic plasticity. This is a major concern of this approach, since the method relies on large numbers of volley stimuli that trigger a postsynaptic action potential. I analyzed spike timings of the postsynaptic cells following volley input, and found that most spikes occurred after 30 ms (Figure 7F). There was little overlap with standard STDP curves [28]–[30]. Based on the shape of experimental curves, I ignored the tail of the STDP curve beyond 30 ms. Taking the product of STDP with the number of spikes for the region before 30 ms, I found that the cumulative amount of potentiation was ∼12% for the case without background activity (0 Hz). Furthermore, these paired spikes could be spread out over a 2-hour period if the block stimuli were interleaved. Hence there would be considerable intervening uncorrelated activity which may act to restore the synaptic weight toward its set point [31]. I also analyzed spike timings with the 10 Hz background, and in this case the overlap was about twice as large and potentially more likely to introduce plasticity. However, the high background activity may again serve to balance out the plasticity over long periods.

### Scalability

The final step in the study was to analyze the scalability of the approach, assuming idealized optical recording capabilities. A specific target was to design the most informative 2-hour slice recording experiment. The design for this experiment was constrained by the characteristics of optical stimulation and recording, by neuronal projection patterns, by plasticity, and by the number of trials needed to build up statistical confidence. Plasticity effects are likely to be relatively small, as calculated above. I consider the number of trials here, and the remaining points in the discussion section.

**Figure 8:**
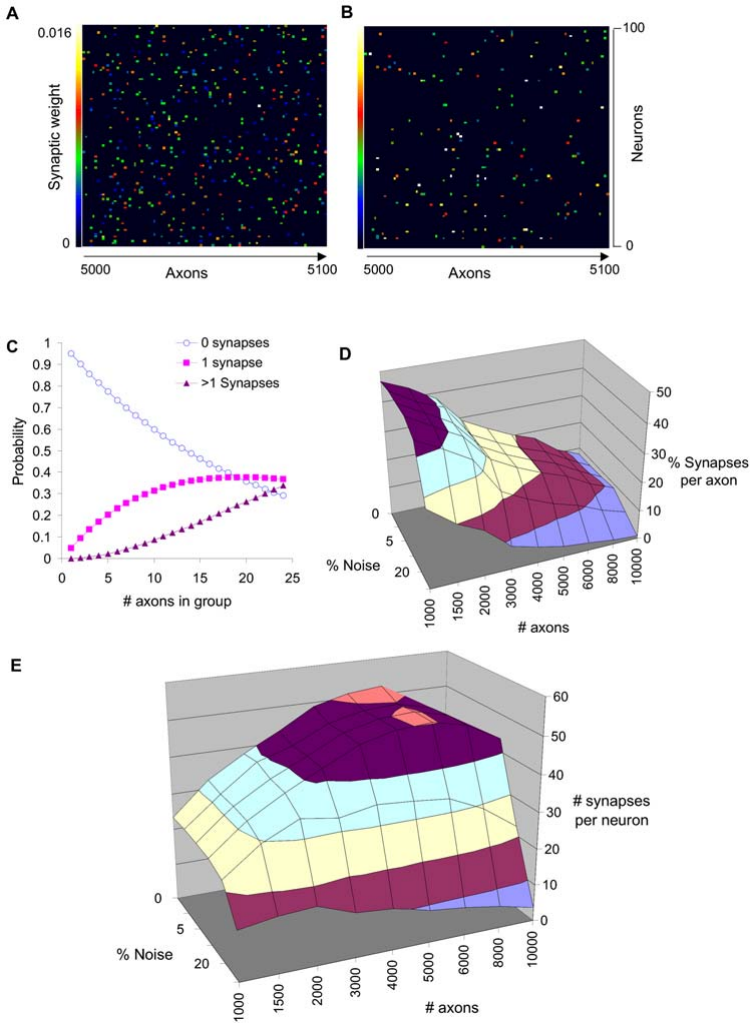
Method Scaling. (A) Actual synaptic weight matrix for axons 5000 to 5100. (B) Predicted synaptic weight matrix for same axons, using 20% noise and 80 repeats and regression fit as in (6D). Most of the low-weight synapses are missing. (C) Grouping axonal stimuli, with connection probability = 5%. More synapses were connected as the number of axons in the group increased, but the number of ambiguous cases with more than one synapses also rose. (D) Percent of synapses reported per axon in a 2-hour experiment at 300 ms per trial, with less than 1% false positives out of the reported synapses. Almost 50% of synapses were found for 1000 axons, as there was time for 240 repeats. With more axons, only a few trials were possible per axon and the percentage declined. Synapse identification fell sharply at 50% noise. (E) Number of synapses found per neuron in the same 2-hour experiment. More axons improved input coverage, but the number of possible trials decreased. There was a peak of ∼50 detected synapses per neuron, for ∼6000 axons.

As a baseline for this analysis, I considered the synapse selectivity achieved so far. Out of a possible 1 million synaptic contacts (10,000 inputs and 100 output neurons) the actual simulated circuit had ∼50,000 synapses, of which ∼11,000 (about 22%) were resolved using the modified KS method. Most of these were the strong synapses (Figure 8A, B). In separate simulations using 10Hz background activity the method gave ∼35% coverage of synapses (Figure 7E). However, both these simulated experiments required too many stimulus trials to be practical.

I analyzed the tradeoffs between number of trials, statistical confidence, and number of stimulated axons. To improve the detection of synapses, it was necessary to maximize the number of trials, by minimizing the duration of each stimulus cycle. The 10 ms interval between stimulus pulses was close to the minimum set by ChR2-stimulated firing rates [6], and also by the dynamics of synaptic priming and release [23]. This set 60 ms as the shortest time for the 7 probe pulses. Following these pulses, the Ca^2+^ response itself took ∼150 ms to complete (Figure 1). To allow for some settling, I considered total trial durations of 300 ms.

I analyzed a tradeoff that could increase the number of synaptic measurements by an order of magnitude. I considered supra-minimal electrode stimulation of X probe neurons, or equivalently, optical stimulation of groups of X neurons expressing ChR2. A stimulus would be unambiguous if there were either zero or one synaptic contacts per CA1 neuron out of this set of X axons (Figure 8C). For 5% synaptic connectivity, and 10% ambiguous synapses, X could be as large as ten axons.

I analyzed an optimally designed experiment of 2 hours, grouping probe axons into sets of ten as described above. I scaled the number of stimulus repetitions inversely with the number of axonal probes, so as to retain the same total experiment time. To do so I performed additional simulations with up to 240 repetitions, on a reduced network with the same 10,000 inputs but only 12 CA1 neurons because of computational limitations. I used the appropriate number of trials taking samples from the 240 and 80 repetition cases for subsequent calculations. In each case I stipulated that the number of false positives was less than 1% of the number of reported synapses. I first analyzed how the fraction of reported synapses per axon scaled with noise and number of axons (Figure 8D). Nearly 50% of synapses were identified for 10% noise and 1000 axons. I then considered how many synapses were reported per target neuron (Figure 8E). Here, the coverage of potential synapses rises with number of axons, but because of time limitations the number of repeats falls with greater numbers of axons. Overall, the number of synapses per neuron peaked at about 50 synapses for 6000 stimulating axons. Thus a 2-hour experiment, recording from 10,000 neurons with 10% instrumentation noise should resolve approximately 500,000 synapses.

## Discussion

This study shows that single synaptic inputs can modulate a suprathreshold background input to produce a measurable shift in the distribution of action potential firings, and consequent calcium transients. The method relies on stochastic resonance between the noisy baseline synaptic input and sub-threshold synaptic events, and generates a readout of action potentials which can be monitored using extracellular electrodes or calcium recordings. Current electrical and optical methods should already be technically capable of using such shifts to record hundreds of single synaptic weights. This study further predicts that new optical stimulation and optical recording methods may be deployed to obtain very large connectivity matrices with single-synapse resolution.

### Testing the Method

In order to validate the proposed approach, a conclusive experimental method for identifying synapses must be combined with this high-throughput experimental analysis. One possible experimental design would be to perform patch recordings in conjunction with bipolar electrode stimulation and dye recording from the target patched neuron. The patch recordings would detect putative single synaptic inputs to compare with the statistical analysis from the optical recording method. Paired patch recordings may be necessary to show that the input is from precisely one neuron. This experiment would allow us to test if the predicted true and false positives are as accurate as these simulations suggest.

### How Informative Is Partial Coverage?

Using this approach we can at best sample from about 50 synapses (<1%) per neuron, from perhaps 10,000 neurons in a slice (∼2% of hippocampal CA1 neurons) [32],[33]. How useful is such a sparse sampling of synaptic connectivity? While it is difficult to anticipate outcomes of these proposed experiments, there are grounds to expect that even a sparse functional wiring diagram would be very informative. First, known hippocampal representations of space are distributed and broad [34]. Recent direct experiments on hippocampal memory indicate that some aspects of memory traces may be observed even from a small number of recording electrodes [35]. Thus a sparse sample may cover a substantial number of synapses involved in ‘memory engrams’. Second, even a sparse circuit measurement may reveal signatures of repeated neuronal microcircuits (e.g.,[3],[12],[36]). Indeed, almost all current knowledge of vertebrate circuitry has been obtained from sparse sampling methods combined with neuroanatomy [1],[32]. Third, the coupling of precise but sparse functional data with new anatomical methods such as block-face sectioning [37] and multicolor genetic labeling [38] may build a more complete picture of neural circuitry than either approach on its own. Such a combination is especially important because geometrical connectivity does not always translate to functional connectivity [39].

### Other Systems

This analysis was done on the relatively simple neuronal circuit in the CA1, and ignores interneurons. Other brain regions with more complex circuits will require their own stimulus designs and the deployment of multiple kinds of optogenetic or electrical stimuli. The KS analysis should be effective for inhibitory as well as excitatory inputs, but would not work well for weak synapses (Figure 6, 8).

In many neuronal circuits (e.g., cortex) there are many local circuits in addition to long-range fiber tracts. In such cases, interneurons and recurrence complicate the analysis, which is too slow to resolve polysynaptic effects. Our preliminary calculations (Fig 7) suggest that the method may be able to resolve monosynaptic input in the presence of as much as 60% recurrence. Nevertheless, it will require a cortex-specific study to better understand the capabilities of this approach in the far more complicated cortical circuit. A possible experimental approach to reversibly ‘simplify’ such networks is to transiently silence interneurons using pharmacological blockers or halorhodopsin [40].

While the current analysis assumes the use of brain slices, the general multi-input/multi-output approach is readily carried over to in-vivo recordings. Our data suggest that modest levels of background activity would be tolerated by the method. Optical methods have already been employed in vivo [5], and electrode recordings routinely monitor hundreds of neurons [18]. Electrode recordings have the additional advantage of fine time resolution, which allows the use of spike-timing data that was discarded in this study.

### Scalability and Technology

The fundamental benefit as well as difficulty of this approach is its scalability. The benefit is that the number of monitored synapses scales as the product of recorded and stimulated neurons. The difficulty is due to the increasingly stringent timing and accuracy requirements at larger scales. Current array electrodes have ∼60 contact points [9], each of which could be used with near-minimal stimulation to address a set of around 10 axons. Current optical methods can readily record from 100 individual cells in the slice [17]. With the assumptions of 5% connectivity and a 50% synapse detection rate, it should be possible to record from ∼1500 synapses in this configuration. This contrasts favorably with the current maximum of ∼12 patch electrodes, which should yield about 6 synapses assuming 5% connectivity. Beyond these current capabilities, a major goal of this study was to extrapolate from existing methods and set technical targets that would enable high-throughput recording.

The slice configuration itself would require some optimization. Neuronal projection patterns in the hippocampal slice are well known. With careful selection of the plane of slicing, it is possible to establish unbroken connections between CA3 and CA1 neurons. Nevertheless, it is challenging to retain enough connections to achieve several thousand intact axonal projections.

Both stimulation and recording may require optical techniques to scale up to very large network reconstructions. Methods already exist to do so for up to 1000 neurons in cortex [41]. In the hippocampus one may have to use separate CA3 stimulation and CA1 recording scanning optics. Two-photon methods are likely to be required for sufficient resolution in each case. This is currently feasible for the recordings, and for glutamate uncaging, but to my knowledge two-photon stimulation of optogenetic constructs is yet to be demonstrated. One possible configuration may use paired inverted and upright optical assemblies. Another possible configuration could utilize light guides [42] to provide the stimulus.

These simulations suggest a target of ∼80 repeats per input to achieve around 20% accuracy in synaptic identification. To deliver the required stimulus, the stimulating apparatus must generate reliable action-potential trains with ∼10 ms resolution, applied to ∼10,000 CA3 neurons. While this level of accuracy has been achieved with illumination of single neurons [40], it will require fast and precise scanning methods [43] to illuminate many neurons within this time window. The block design of the method relaxes these constraints significantly, so that only a handful (one to 10) axons/neurons must be stimulated precisely (within a ∼10 ms window) for any given trial, and the rest can be activated together using broad illumination or supraminimal electrical stimulation. In sum, the stimulus scaling targets appear achievable.

The technical issues with scaling up the number of recorded CA1 neurons are familiar ones of scanning speed versus signal-to-noise versus photobleaching. The suggested 2-hour experiments are feasible for a small number of neurons without much photobleaching using enhanced CCD cameras (Parameshwaran and Bhalla, unpublished data). It is more challenging to perform long recordings using 2-photon imaging, but improved calcium reporters may extend the duration of such recordings as well. There are already Ca^2+^ recording methods which can monitor ∼1000 individual neurons [17]. This suggests that it should be possible to scale up recordings to several thousands of neurons. Accurate algorithms for estimating spike counts from experimental Ca^2+^ waveforms have been developed [44],[45], and these may give higher classification accuracy with better noise immunity that the methods in this study. Combining ∼50-synapses per neuron (Figure 8E), and ∼10,000 recorded neurons, the target of almost a million synapses in a 2-hour experiment should be an ambitious but achievable technical goal. Such data would be a significant step toward reconstructing the functional wiring diagram of large neuronal circuits.

## Methods

### Compartmental Modeling

Input (CA3) neurons were modeled as single compartment passive cells with a spiking threshold and a 2 ms refractory period. Inputs were provided as a brief (60 microsecond) current pulse to represent electrical stimulation, but were also tested to give equivalent spiking output with smaller but longer current pulses representing light input to ChR2. Output (CA1) neurons were modeled as 19-compartment neurons slightly modified from Traub et al. [46] with the inclusion of NMDA and AMPA receptors. These neurons included Na, K, K_Ca and L-type Ca^2+^ channels and incorporated simple pump-based Ca^2+^ dynamics (Figure 1, Dataset S2). The time-courses of calcium in these cell models are too fast, for two reasons. First, the modeled neurons are based closely on models by Traub et al. [46], which use relatively rapid Ca^2+^ kinetics. Second, experimental recordings use Ca^2+^ indicators, which act as chelators and are therefore relatively slow. Optical recordings from CA1 somas have time-courses of the order of 150 msec (Parameshwaran, Madhavan, and Bhalla, unpublished data). However, the calcium time-course should not affect these calculations, because the analysis is based on the total area of the calcium transient.

### Network Parameters

Input axons were connected onto the NMDA and AMPA receptors of the CA1 neurons using a 5% connection probability [22]. Synaptic weights were set up using one of two Gaussian-based distributions: 1. A flat distribution with an upper cutoff (standard deviation = 1.0, upper cutoff = 1.0 standard deviations). 2. A narrower distribution with standard deviation = 0.5 and upper cutoff = 2.0 standard deviations, based on synaptic area estimates of Sorra and Harris [27]. The mean weight was set to 0.006 (arbitrary units) so as to give the response profile in Figure 1, where approx. 50 inputs were required to elicit an action potential. The requirement to keep this number of inputs around 50 meant that the synaptic conductances were somewhat smaller than estimated for CA1 synapses, because of the short length constant of the single simulated apical dendrite. The peak synaptic conductance reached following input on a single synapse was:
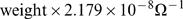
(See Dataset S2.) I used a known random number seed for the network setup, so as to generate the same weight matrix to compare across many simulations. I used the same weights for NMDA and AMPAR conductances, but if the AMPA conductance was less than half of the mean it was set to zero to represent silent synapses.

I used two readouts for the Ca^2+^ response: (1) the area under the curve of the Ca^2+^ signal from 10 to 300 ms; (2) the time of the first Ca^2+^ transient, measured as time when the Ca^2+^ signal crossed a preset threshold.

Most runs used 10,000 CA3 neurons as inputs, and 100 CA1 output neurons, but for >80 repeats I reduced the model to 12 CA1 neurons because of computational limitations. Action potential propagation velocity was set to 1.0 m/s. The 10,000 Schaffer collaterals were distributed in the proximal 240–740 microns of the dendrite and ran in parallel.

### Neuronal Variability

I modeled variability between cells by scaling key passive and active properties of all neurons in the network using the equation
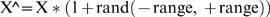
(2)

- Where X is the reference parameter, Xˆ is the randomly altered parameter, and range specifies how much variability to introduce. I used range = 0.2 to obtain ±20% variability, and 0.5 for 50% variability. I used Equation 2 above with a different random number for each parameter in each compartment in each cell in the model. The altered parameters were: Rm, the membrane resistance, and Cm, which was altered in inverse proportion to Rm for the same compartment. This relationship assumed that the biological variability was due to surface area change.
- Ra: Axial resistance
- Gmax: the channel conductance of every voltage-gated ion channel.

I did not alter the channel kinetics or reversal potentials.

### Synaptic Stochasticity

I modeled stochastic synaptic transmission using a simple Monte Carlo method based on measured release probabilities and facilitation [23]. I used the presence of inputs to the model CA3 neuron as a surrogate for probabilistic synaptic release on all synapses on the axon of that neuron. Different CA3 neurons used independent release calculations. I triggered stimuli with a 40% probability on the first pulse. After the first pulse, synaptic release probability was 90% if no release had yet occurred. If one or more releases had occurred, synaptic release probability was 55%. Note that the entire simulated axon was triggered with this probability, though in reality the individual synapses should function independently. This simplification should not affect the primary results as the CA1 neurons are independent in most of the calculations. In the recurrent circuits there may be some effects of this correlation across inputs but it is unlikely to affect synaptic detection.

### Readout Noise

This was added to all Ca^2+^ responses as a Gaussian distribution with a mean of zero, and a standard deviation set to the desired scaling factor. The random number generator was the Mersenne Twister [47].

### Simulation Environment

All simulations were run using the GENESIS simulator [48] using a 50 microsecond timestep. Large calculations were run on a 260-CPU cluster of Opteron processors (Sun microsystems/Locus computing) running the Linux operating system. The simulation source files are provided as Dataset S3.

### Statistical Analysis

To analyze the responses I looked for differences between responses for each individual block+probe response vs. the combined responses for the entire block, as a reference. As an initial analysis I used means and standard errors of each of these distributions. Given the strongly non-normal distribution of responses, I then used the Kolmogorov-Smirnov (KS) test.

For the mean/SEM analysis, I used two parameters to tune the sensitivity:
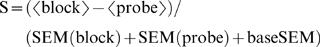
(3)I categorized a response as due to a synapse if S was *greater* than a threshold.

Here baseSEM was the first parameter, and the threshold the second.

For the KS test, I used the standard incomplete gamma function estimator (Q) for probability of obtaining the observed difference between baseline and probe distributions. I categorized a response as due to a synapse if the probability P was less than the threshold.

In both cases I set the threshold according to the criterion that less than 1% of the identified synapses should be false positives. The 1% false positive rate was picked as a conservative cutoff, because in circuit reconstruction false positives would be more problematic than false negatives.

This meant that the threshold had to be adjusted depending on the number of reported synapses.

The KS test provided a P value which mapped to the number of false positives more consistently than the 2-parameter mean/SEM test. The modified KS test also worked consistently with the inclusion of a scale factor:
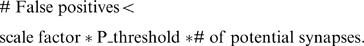
(4)This equation made it possible to obtain a good estimate of false positives, and hence to maintain accurate synapse selectivity from the data. The only additional datum required was an estimate of the number of potential synapses, which is the product of the synaptic connectivity and the number of stimulated axons. The synaptic connectivity value has been estimated for many systems and is around 5% for CA3 to CA1 projections [22].

I found that a scale factor of 10 was quite conservative. So, for ∼50,000 synapses in the simulations, there should be <80 false positives for a P-threshold of 0.00016. The actual value of false positives for P = 0.00016 was in the range of 30 to 50 for several variants of the model and at several values of instrumentation noise. Based on these estimates, the criterion of under 1% false positives would be met if there were over 8000 reported synapses for a P-threshold of 0.00016.

All statistical tests were custom coded in C++. The implementation of the KS test was based on Press et al. [49].

The original KS test was too sensitive to instrumentation noise. For extremely low noise the KS test gave very good results, but for even moderate levels of noise the test failed. This was because the algorithm was classifying responses based on subtle differences in peak amplitudes rather than on the number of action potentials. I therefore implemented a variant on the KS test that selected cases where the difference between the distributions spanned a wide response amplitude range (Figure S2). The specific modification to the KS test was that the maximum vertical difference used for the test should only be considered if the difference between the distributions had the same sign over a certain minimum amplitude (x-axis) range. This x-axis range had a value of 1.0+10% of the maximum amplitude in the distribution. For comparison, typical single calcium spikes had an amplitude of ∼10 units. Overall, this modification biased the KS statistic toward robust and large shifts in Ca^2+^ signal, such as might be expected for different numbers of action potentials.

I also tested how to combine responses for the same probe when it was stimulated along with different background blocks. I tried several ways of combining such responses, including taking logical combinations (AND and OR) of individual probe classifications, and summing the P or S values from the individual probes. Although combining probe information usually did improve synaptic classification, the improvement was less than simply running twice as many repeats on the same probe (data not shown). So the most economical way of obtaining good classifications seemed to be to simply use a single probe position.

## Supporting Information

Dataset S1 Source code for Monte Carlo calculations for synaptic input distributions.
(0.05 MB TAR)

Dataset S2 Model parameters.
(0.03 MB PDF)

Dataset S3 Simulation source files.
(0.07 MB TAR)

Figure S1 Noise-free synaptic estimation.
(0.03 MB PDF)

Figure S2 Kolmogorov-Smirnov analysis of calcium signals.
(0.04 MB DOC)

Video S1 Video of reduced network responding to baseline and baseline+probe stimuli.
(16.84 MB MOV)
