# Supplementary material for: How To Record a Million Synaptic Weights in a Hippocampal Slice
Source: PLoS Comput Biol. 2008 Jun 20;4(6):e1000098. doi: 10.1371/journal.pcbi.1000098 (PMC2409153; doi:10.1371/journal.pcbi.1000098)
Supplement: Dataset S2 — Model parameters. (0.03 MB PDF) [file pcbi.1000098.s002.pdf]

CA3 model constants

All voltages are in V.

EREST	-0.07	
Membrane parameters:		
RM (ohm.m^2)	1	
RA (ohm.m)	1	
CM (F/m^2)	0.01	
Spiking threshold	-0.02	
Absolute refractory period	2 ms	
Compartment name	Length (um)	Diameter (um)
soma	20	20

CA1 model constants

All voltages are in V.

EREST	-0.06
ENa	0.055
EK	-0.075
ECa	0.08
Membrane parameters:	
RM (ohm.m^2)	1
RA (ohm.m)	1
CM (F/m^2)	0.03

We use the Hodgkin-Huxley form for gating of a voltage-gated channel:
g = gmax * m^x * h^y
where m and h are gating variables, and x and y are powers.
m and h obey differential equations of the form:
dm/dt = m_alpha * (1 - m) - m_beta * m
In the equations below, this differential form is assumed for all gates unless stated otherwise.

Voltage-gated Ca channel

g = gmax * s^2 * r	A	B	C	D	F
s_alpha = (A + B * x) / (C + exp((x + D) / F))	1600	0	1	-0.005	-0.01389
s_beta = (A + B * x) / (C + exp((x + D) / F))	178	2.00E+04	-1	-0.0089	0.005

r_alpha = 5 * exp(-50 * (V + 0.06)) for V > -0.06
r_alpha = 5 for V < -0.06

Ca concentration pool (note that units are NOT uM)
d(conc)/dt = sum(Ca_influx) - conc / 0.01333

K_AHP

g = gmax * z
z_alpha = 0.02 * conc   for conc < 250
z_alpha = 10.0   for conc >= 250

z_beta = 1.0 + 0.02 * conc   for conc < 250
z_beta = 11.0   for conc >= 250

K_C

g = gmax * x * z
for V < -0.01:
x_alpha = exp (53.872*(V - EREST) - 0.66835)/0.018975
x_beta = 2000*exp((EREST + 0.0065 - V)/0.027) - x_alpha(V)
for V > -0.01:
x_alpha = 2000*exp((EREST + 0.0065 - V)/0.027)
x_beta = 0

The z gate is computed directly from Ca concentration, not using a differential equation:
For Ca < 125:
z = Ca * 0.008
For Ca > 125
z = 1

Na

g = gmax * m^2 * h	A	B	C	D	F
m_alpha = (A + B * x) / (C + exp((x + D) / F))	-15008	3.20E+05	-1	0.0469	-0.004
m_beta = (A + B * x) / (C + exp((x + D) / F))	5572	2.80E+05	-1	0.0199	0.005
h_alpha = (A + B * x) / (C + exp((x + D) / F))	128	0	0	0.043	0.018
h_beta = (A + B * x) / (C + exp((x + D) / F))	4000	0	1	0.02	-0.005

K_DR

g = gmax * n	A	B	C	D	F
m_alpha = (A + B * x) / (C + exp((x + D) / F))	-398.4	-1.60E+04	-1	0.0249	-0.005
m_beta = (A + B * x) / (C + exp((x + D) / F))	250	0	0	0.04	0.04

**GluR channel**	τ1	τ1	
$g_{syn}(t) = (A.g_{max}./(\tau1 - \tau2)).(\exp(-t/\tau1) - \exp(-t/\tau2))$	2 ms	9 ms	
A is a normalization constant obtained as follows:			
$\tau pk = \tau1 * \tau2 * \ln(\tau1 / \tau2) / (\tau1 - \tau2)$			
$A = (\tau1 - \tau2) / (\tau1 * \tau2 * \exp(-\tau pk / \tau1) - \exp(-\tau pk / \tau2))$			
**NMDAR channel: conductance term**			
$g_{syn}(t) = Block.(A.g_{max}./(\tau1 - \tau2)).(\exp(-t/\tau1) - \exp(-t/\tau2))$	20 ms	40 ms	
NMDAR channel: Mg block term			
	eta	gamma	[Mg]
Block = 1/eta * (1/eta - [Mg]*exp(-V * gamma))	0.28	62	1.2

NMDAR channel: Ca influx term. This is computed separately from the conductance term using identical equations but using Eca = 0.1 V which differs from the current reversal potential. We assume that Ca carries 1/10 of the NMDA channel conductance.

Cell model compartmental structure

Compartment name and type	Length (um)	Diameter (um)	Channel densities (Ohm^{-1}/m^2) Ca	K_AHP	K_C	Na	K_DR	Glu	NMDA
Apical_19:	120	5.78	0	0	0	0	0	10	4
Apical_18:	120	5.78	50	8	50	0	0	10	6
Apical_17:	120	5.78	50	8	50	0	0	10	8
Apical_16:	120	5.78	50	8	50	0	0	10	8
Apical_15:	120	5.78	70	8	50	0	0	10	8
Apical_14:	120	5.78	70	8	50	0	0	10	10
Apical_13:	120	5.78	70	8	50	0	0	10	10
Apical_12:	120	5.78	170	8	150	200	200	10	10
Apical_11:	120	5.78	50	8	50	0	50	10	10
Apical_10:	120	5.78	80	8	200	150	100	10	10
Soma:	125	8.46	40	8	100	300	250	12	0
Basal_8	110	4.84	80	8	200	150	100	10	0
Basal_7	110	4.84	50	8	50	0	50	10	0
Basal_6	110	4.84	120	8	100	200	200	10	0
Basal_5	110	4.84	70	8	50	0	0	10	0
Basal_4	110	4.84	70	8	50	0	0	10	0
Basal_3	110	4.84	50	8	50	0	0	10	0
Basal_2	110	4.84	50	8	50	0	0	10	0
Basal_1	110	4.84	0	0	0	0	0	10	0
