# Supplementary material for: How To Record a Million Synaptic Weights in a Hippocampal Slice
Source: PLoS Comput Biol. 2008 Jun 20;4(6):e1000098. doi: 10.1371/journal.pcbi.1000098 (PMC2409153; doi:10.1371/journal.pcbi.1000098)
Supplement: Figure S1 — Noise-free synaptic estimation. (0.03 MB PDF) [file pcbi.1000098.s004.pdf]

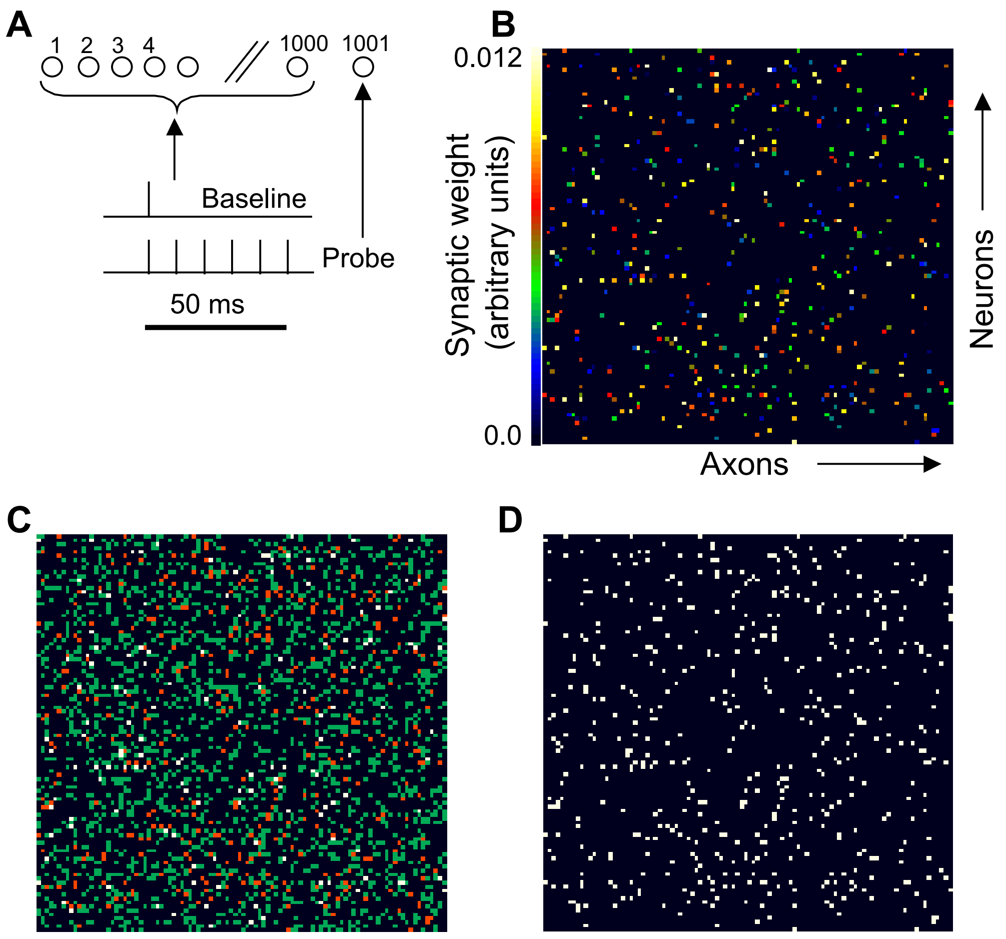

Supplementary figure S1. Noise-free synaptic estimation. (A) Stimulus design. The first 1000 axons in a block were used for the baseline input, consisting of a single action potential with 100% release probability. The 1001st axon was used for the probe stimulus consisting of 6 action potentials 10 ms apart. The positions of the baseline and probe were shifted by one axon at a time to work around the entire set of axons. (B) Color-coded synaptic weight matrix, showing first 100 axons out of 10,000. Black means no synapse. Synaptic connectivity was 5%. (C) Predicted synaptic weight matrix, with 0.5% readout noise. Black is true negative, red is false negative, green is false positive, white is true positive. The accuracy was poor. (D) Predicted synaptic weight matrix with zero readout noise was nearly perfect.
